# Argonaute-2 protects the neurovascular unit from damage caused by systemic inflammation

**DOI:** 10.1186/s12974-021-02324-7

**Published:** 2022-01-06

**Authors:** Marta Machado-Pereira, Cláudia Saraiva, Liliana Bernardino, Ana C. Cristóvão, Raquel Ferreira

**Affiliations:** 1 Health Sciences Research Centre (CICS-UBI), University of Beira Interior, Rua Marquês d’Ávila e Bolama, 6201-001 Covilhã, Portugal; 2 NeuroSoV, UBImedical, EM506, University of Beira Interior, Covilhã, Portugal; 3 Present Address: Luxembourg Centre for Systems Biomedicine (LCSB), University of Luxembourg, 7 Avenue des Hauts-Fourneaux, 4362 Esch-sur-Alzette, Luxembourg; 4 CEDOC, NOVA Medical School|Faculdade de Ciências Médicas, Universidade NOVA de Lisboa, Campo dos Mártires da Pátria, 130, 1169-056 Lisboa, Portugal

**Keywords:** Argonaute-2, Lipopolysaccharide, Brain endothelial cells, Glia, Secretome, Neuroprotection

## Abstract

**Background:**

The brain vasculature plays a pivotal role in the inflammatory process by modulating the interaction between blood cells and the neurovascular unit. Argonaute-2 (Ago2) has been suggested as essential for endothelial survival but its role in the brain vasculature or in the endothelial–glial crosstalk has not been addressed. Thus, our aim was to clarify the significance of Ago2 in the inflammatory responses elicited by these cell types.

**Methods:**

Mouse primary cultures of brain endothelial cells, astrocytes and microglia were used to evaluate cellular responses to the modulation of Ago2. Exposure of microglia to endothelial cell-conditioned media was used to assess the potential for in vivo studies. Adult mice were injected intraperitoneally with lipopolysaccharide (LPS) (2 mg/kg) followed by three daily intraperitoneal injections of Ago2 (0.4 nM) to assess markers of endothelial disruption, glial reactivity and neuronal function.

**Results:**

Herein, we demonstrated that LPS activation disturbed the integrity of adherens junctions and downregulated Ago2 in primary brain endothelial cells. Exogenous treatment recovered intracellular Ago2 above control levels and recuperated vascular endothelial-cadherin expression, while downregulating LPS-induced nitric oxide release. Primary astrocytes did not show a significant change in Ago2 levels or response to the modulation of the Ago2 system, although endogenous Ago2 was shown to be critical in the maintenance of tumor necrosis factor-α basal levels. LPS-activated primary microglia overexpressed Ago2, and Ago2 silencing contained the inflammatory response to some extent, preventing interleukin-6 and nitric oxide release. Moreover, the secretome of Ago2-modulated brain endothelial cells had a protective effect over microglia. The intraperitoneal injection of LPS impaired blood–brain barrier and neuronal function, while triggering inflammation, and the subsequent systemic administration of Ago2 reduced or normalized endothelial, glial and neuronal markers of LPS damage. This outcome likely resulted from the direct action of Ago2 over the brain endothelium, which reestablished glial and neuronal function.

**Conclusions:**

Ago2 could be regarded as a putative therapeutic agent, or target, in the recuperation of the neurovascular unit in inflammatory conditions.

**Supplementary Information:**

The online version contains supplementary material available at 10.1186/s12974-021-02324-7.

## Background

The brain vasculature exhibits unique properties that are required to coordinate parenchymal homeostasis and provide protection from circulating pathological elements (e.g., viruses, bacteria, toxins). The inner walls of blood vessels are lined by endothelial cells, which mediate the interactions between the bloodstream and neural cells, becoming a key component in blood–brain barrier (BBB) function and integrity [1]. Understanding these cellular interactions is pivotal to disclose the mechanisms behind BBB disruption occurring in brain disorders and pathologies, and to promote vascular recovery. Cell activation unleashed by systemic inflammation is conducive to the generation of a pool of circulating mediators, i.e., cytokines, growth factors and vasoactive molecules that stimulate cell recruitment and adhesion, and sustain further cell activation, which may impair BBB function and aggravate tissue damage [2, 3].

Considering that a major anti-angiogenic factor is inflammation, we focused on the endothelial–glial crosstalk, using the endotoxin lipopolysaccharide (LPS) as the inflammatory cue. LPS has a direct effect on endothelial cells, namely on vasoregulation, vascular permeability, leukocyte recruitment and adhesion, with most work focusing on cell lines and/or peripheral vessels [4]. LPS is a classical inducer of Toll-like receptor 4 (TLR4) signaling [5]. The TLR4 pro-inflammatory pathway requires tight control to avoid cytokine-related cell death and tissue damage and, in that sense, uses microRNA (miRNA) as a mechanism for negative regulation [6]. These small but impactful non-coding molecules interact with messenger RNA and repress protein synthesis [7, 8]. Argonaute-2 (Ago2) is one of the key participants in the canonical biogenesis of miRNA, with the assistance of chaperones HSC70 and HSP90 [9]. Although there are other members of the Argonaute family, Ago2 is the only human Ago protein with endonuclease activity. In other mammals, the non-catalytic Ago proteins (Ago1, Ago3 and Ago4) can act redundantly to some extent [10]. Additionally, it operates as a natural carrier: most miRNA in the human plasma circulate in Ago2 ribonucleoprotein complexes [11, 12], suggesting both intracellular and extracellular levels of Ago2 matter. Indeed, Ago2 knockdown directly correlates with a decrease in the expression of mature miRNA [13], while its upregulation is associated with a global increase [14, 15]. In a non-pathological vascular context, Ago2 knockdown per se compromised cell survival and the formation of tubules (primitive vascular network) by human umbilical vein endothelial cells (HUVEC) [16], indicating a role in angiogenesis. Moreover, Ago2 downregulation specifically decreased vascular endothelial growth factor (VEGF) expression and signaling in hepatocellular carcinoma cell lines (Huh7 and SMMC-7721). The infection with a recombinant adenovirus expressing Ago2 restored VEGF expression and release in these cells [17]. Finally, we reported that Ago2 facilitates miR-18a entry into human and mouse brain endothelial cells in vitro and in vivo [18], respectively. We found that brain endothelial cells were highly permissive to miRNA uptake compared to other cell types, and uptake depended on Ago2 concentration. Extracellular Ago2 can be internalized through neuropilin-1 (NRP1) [19]. Neuropilins also bind to other ligands, such as class III semaphorins and members of the vascular endothelial growth factor (VEGF) family [20]. However, we did not discriminate against the impact of specific levels of Ago2 on brain endothelial cells under pathological or inflammatory conditions.

In this work, our aim was to clarify the role of Ago2 in (i) brain endothelial cells and glia function under inflammatory conditions induced systemically, and (ii) in the repair of inflammation-afflicted neural tissue. Herein, we demonstrated that LPS downregulated Ago2 and increased permeability of brain endothelial cells in vitro, while Ago2 treatment restored these parameters. The conditioned media from Ago2-restored endothelium preserved the activity of microglia. Activated microglia overexpressed Ago2 and Ago2 silencing partially contained the inflammatory response, but LPS challenge did not affect astrocytic Ago2. It was only necessary to maintain the basal release of tumor necrosis factor (TNF)-α in vitro. In vivo, the administration of Ago2 in LPS-injected animals produced a protective response in the neocortex and hippocampus, by reversing endothelial activation and securing normal glial and neuronal function. Therefore, Ago2 could be used as a potential agent for intravascular therapies, or a target for deactivating microglia, in an inflammatory setting. Considering the importance of inflammation in neuropathological contexts, further studies should explore the role of Ago2 in the central nervous system.

## Methods

### Primary endothelial cell cultures

Mouse brain endothelial cells were isolated using a protocol adapted from [21]. Briefly, the cortices of 3- to 7-day-old C57BL/6J pups were isolated, minced and resuspended in 0.25% trypsin (Gibco, Barcelona, Spain). Then, tissue suspension was centrifuged at 350*g* for 5 min. The pellet was filtered through a 70-μm nylon cell strainer (Falcon, Corning Incorporated, NY, USA) to retain the microvessel fraction, which was seeded onto 0.5% gelatin-coated surfaces (Sigma, MO, USA). Puromycin (0.4 μg/ml; Sigma) was added for selective endothelial cell growth. Brain endothelial cells were maintained at 37 °C in a 95% atmospheric air and 5% CO_2_ humidified atmosphere in Eagle’s minimum essential medium (Lonza, MD, USA) supplemented with 100 U/ml penicillin and 100 μg/ml streptomycin (Life Technologies, Barcelona, Spain), 10% fetal bovine serum (Millipore, Berlin, Germany), 10% horse serum (Thermo Fisher Scientific, MA, USA), 10 μg/ml epidermal growth factor (Invitrogen, CA, USA), 100 μg/ml heparin sodium salt (PanReac AppliChem, Barcelona, Spain) and 2 mM glutamine (Sigma). Culture purity is above 95%, measured by immunocytochemistry (data not shown). Cells were plated at a density of 2.5 × 10^4^ cells per well in 24-well trays (immunocytochemical studies), 1 × 10^5^ cells per well in 6-well trays (Griess assay, enzyme-linked immunosorbent assays and western blot analysis), or 7.5 × 10^3^ cells per well in 96-well trays (cell viability assays). Cell treatments included LPS stimulation (100 ng/ml; Sigma) and/or Ago2 administration (0.4 nM; Abcam, Cambridge, UK) for 24 h and bafilomycin A1 (BafA1; 100 nM; VIVA Bioscience Ltd., Devon, UK) for 3 h. Ago2 silencing is described in a section below. Untreated cells were used as the control condition (CTR).

### Endothelial cell-conditioned media (EC-CM) collection

**Fig. 1:**
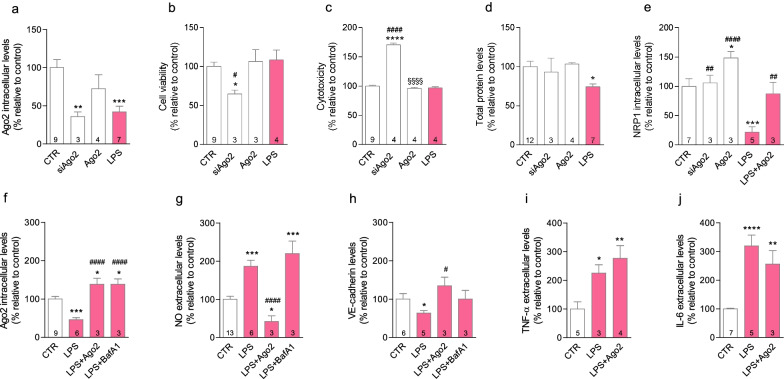
LPS-induced Ago2 downregulation correlates with loss of endothelial function. Brain endothelial cells stimulated with lipopolysaccharide (LPS, 100 ng/ml), for 24 h, exhibited endogenous Ago2 levels significantly lower than untreated cells (control, CTR). This effect was similarly obtained with Ago2 silencing (0.05 µM). Ago2 treatment per se did not change intracellular levels (**a**). Ago2 silencing compromised cell survival (**b**) and induced cytotoxicity (**c**). LPS only caused loss of protein content (**d**). Ago2 treatment had no effect on any of these parameters associated to cell survival (**b**–**d**). While siAgo2 had no effect on NRP1 expression, the receptor was downregulated by LPS and upregulated by Ago2 treatment alone. Ago2 and LPS co-administration maintained NRP1 expression (**e**). Ago2 co-treatment (0.4 nM) restored its intracellular levels in LPS-activated endothelial cells. The same result was produced by autophagy inhibition with bafilomycin A1 (bafA1; 100 nM), measured by western blotting (**f**). LPS-activated cells released nitric oxide (NO) and Ago2 treatment significantly reverted LPS-induced increase of NO levels, measured by Griess assay (**g**). LPS-activated cells showed a decrease in VE-cadherin expression and Ago2 treatment maintained the levels of this intercellular junction protein, measured by western blotting (**h**). LPS-activated brain endothelial cells released pro-inflammatory factors, such as tumor necrosis factor-alpha (TNF-α) and interleukin-6 (IL-6), the replenishment of Ago2 intracellular levels failed to normalize the levels of these cytokines (**i** and **j**, respectively), measured by ELISA. Data are expressed as the mean ± SEM of the indicated number of repeats and as a percentage relative to untreated controls (*p < 0.05, **p < 0.01, ***p < 0.001, ****p < 0.0001 compared to untreated controls; ^#^*p* < 0.05, ^##^*p* < 0.01, ^###^*p* < 0.001, ^####^*p* < 0.0001 compared to LPS-activated cells; one-way ANOVA for all figures; in **i**, Student’s *t* test was used for the comparison between CTR and LPS). *Ago2* argonaute-2, *BafA1* bafilomycin A1, *IL-6* interleukin-6, *LPS* lipopolysaccharide, *NO* nitric oxide, *NRP1* neuropilin-1, *TNF-α* tumor necrosis factor-alpha, *VE-cadherin* vascular endothelial-cadherin

After cell treatments, the EC-CM were collected and clarified by centrifugation at 14,000*g* for 20 min to remove cell debris and stored at – 80 °C before use. Media were used for quantifying inflammatory mediators (Griess assay and enzyme-linked immunosorbent assays (ELISA) in Fig. 1) and for assessing the impact of the endothelial secretome on microglia cell death and activation (Fig. 4).

### Griess assay

Griess reagents were added to each well following manufacturer’s instructions: 0.1% *N*-1-naphthylenediamine dihydrochloride and 1% sulfanilamide in 5% phosphoric acid (Promega, WI, USA). Nitric oxide (NO) production was determined through the formation and accumulation of the stable metabolite product nitrite (NO_2_) by measuring optical density at 540 nm in an enzyme-linked immunosorbent assay plate reader (SPECTRA max 384 Plus, Molecular Devices). The total amount of protein was quantified using the bicinchoninic acid (BCA) assay (Thermo Scientific, MA, USA) and protein levels were used for normalization.

### Enzyme-linked immunosorbent assay (ELISA)

After cell treatments, media were collected and analyzed according to the manufacturer’s instructions. The following detection kits were used: human Ago2 (MyBioSource, CA, USA), mouse interleukin (IL)-1β, mouse IL-6 and mouse TNF-α (BD Biosciences, CA, USA). Optical density readings at 570 nm were subtracted from optical density readings at 450 nm in an enzyme-linked immunosorbent assay plate reader (SPECTRA max 384 Plus, Molecular Devices). Total protein concentration was determined using the BCA assay (Thermo Fisher Scientific) and was used for normalization.

### Primary glial cell cultures

Microglial cells and astrocytes were isolated using a protocol adapted from [22]. Briefly, the brains of 3–5 days old C57BL/6J pups were isolated, stripped of meninges, minced and resuspended in Dulbecco’s modified Eagle’s medium–high glucose (DMEM-HG; Sigma), with 100 U/ml penicillin and 100 μg/ml streptomycin (Life Technologies), and 10% fetal bovine serum (Millipore). The tissue was mechanically dissociated and filtered through a 70-μm nylon cell filter (Falcon) and centrifuged at 250*g* for 10 min. Cells were then seeded onto 0.01% poly-d-lysine-coated flasks. The medium was changed every 2 days. After approximately 10 days, cultures were shaken overnight to recover the microglia cell fraction, which was centrifuged at 230*g* for 8 min, leaving astrocytes in the adherent monolayer. To recover astrocytes, 0.25% trypsin (Gibco) was added, and the detached cell fraction was collected and centrifuged at 230*g* for 8 min and resuspended in DMEM-HG. The medium was changed the next day and then every 2 days. Culture purity is approximately 95%, measured by immunocytochemistry (data not shown). Microglial cells and astrocytes were seeded separately onto 0.01% poly-d-lysine-coated plates (Sigma) at a density of 2 × 10^4^ cells per well in 24-well trays (immunocytochemical and silencing studies), 5 × 10^5^ cells per well in 6-well trays (Griess assay, enzyme-linked immunosorbent assays and western blot analysis) or 7.5 × 10^3^ cells per well in 96-well trays (cell viability assays). Cells were maintained at 37 °C, in 5% CO_2_ and 95% atmospheric humidified air in DMEM-HG, supplemented with 100 U/ml penicillin and 100 μg/ml streptomycin and 10% fetal bovine serum. Cell treatments included LPS stimulation (100 ng/ml; Sigma) for 24 h and/or Ago2 silencing. Control cells were left untreated.

### Argonaute-2 silencing

For Ago2 silencing, cells were first treated for 6 h (microglial cells and astrocytes) or 5 h (brain endothelial cells) with Lipofectamine-delivered (2 µg/ml; Invitrogen) siAgo2 (50 ng/ml; Ambion, Inc, Thermo Fisher Scientific) or a scrambled miRNA sequence (50 nmol/l; Thermo Fisher Scientific), according to the manufacturer’s instructions. Ago2 downregulation was confirmed by western blotting. After Ago2 silencing, cells were washed with phosphate-buffered saline (PBS) and were either exposed to LPS or left unexposed to activation, in complete media.

### Cytotoxicity

After treatments, media were collected and lactate dehydrogenase (LDH) activity was measured using a cytotoxicity assay (CytoTox 96 Non-Radioactive Cytotoxicity assay, Promega Corporation, MA, USA). Cytotoxicity levels were calculated according to manufacturer’s instructions and normalized to the control condition (untreated cells). Optical density was measured at 492 nm in an enzyme-linked immunosorbent assay plate reader (SPECTRA max 384 Plus, Molecular Devices).

### Cell viability

To assess cell viability, cell counting kit-8 solution (CCK‑8; Dojindo Laboratories, Kunamoto, Japan) was added to cells in the remaining 3 h of treatment. Afterwards, cell viability was determined by measuring optical density at 450 nm in an enzyme-linked immunosorbent assay plate reader (SPECTRA max 384 Plus, Molecular Devices). Data were normalized to the control condition (untreated cells). 

### Immunocytochemistry

Cell cultures were fixed with 2% paraformaldehyde (PFA; Sigma), washed with PBS and blocked with a solution of 3% BSA and 0.1% Tween 20 (Sigma) for 20 min at room temperature to prevent nonspecific binding. Cells were incubated with the primary antibody for 30 min at room temperature followed by overnight incubation at 4 °C. Incubation with the respective secondary antibody was performed at room temperature for 2 h. The antibodies used were rabbit anti-Argonaute-2 (Ago2) (1:200; Cell Signaling, MA, USA), rabbit anti-neuropilin-1 (NRP1) (1:200; BD Biosciences), mouse anti-CD31 (Novocastra, Leica Biosystems, Nussloch GmbH, Germany), rabbit anti-VEGFR2 (Abcam, UK), rabbit anti-ionized calcium-binding adaptor molecule-1 (Iba-1) (1:500; FujiFilm Wako Chemicals, VA, USA), mouse anti-glial fibrillary acidic protein (GFAP) (1:500; BD Biosciences), Alexa Fluor 546 donkey anti-rabbit, Alexa Fluor 488 donkey anti-mouse, Alexa Fluor 488 donkey anti-rabbit (all 1:200; Life Technologies) and Alexa Fluor 594 donkey anti-mouse (1:200; Abcam). The nuclei were stained with Hoechst 33342 (4 μg/ml; Molecular Probes, OR, USA). Cell preparations were mounted in Dakocytomation fluorescent medium (Dakocytomation Inc., CA, USA) and images were acquired with AxioImager A1 microscope (Carl Zeiss, Gottingen, Germany).

### Animal studies

All experiments were performed in accordance with National Institutes of Health and European Convention for the Protection of Vertebrate Animals Used for Experimental and Other Scientific Purposes (European Union directive number 2010/63/EU) for the care and use of laboratory animals. Mice were kept in appropriate cages under temperature-controlled conditions with a fixed 12 h light/dark cycle, with free access to food and water. All efforts were made to reduce the number of animals and to minimize suffering. There were no signs of discomfort or significant loss of weight until the end of the experiments. Adult C57BL6 male mice (20-week-old) were first injected intraperitoneally with LPS (2 mg/kg) [23, 24]. In the following 3 days, mice received one intraperitoneal injection of Ago2 (0.4 nM; Abcam) prepared in saline, per day, as described by us and others [18, 25]. Control groups included animals injected with saline alone (sham controls) or injected with Ago2 alone. The day after the injection protocol was completed, animals were euthanized, and their brains removed for western blotting analysis (injection protocol depicted in Fig. 5m). For the immunohistochemistry protocol, mice were anesthetized with an intraperitoneal injection of xylazine (10 mg/kg of mouse weight; Rompun 2%, Bayer, Germany) and ketamine (90 mg/kg of mouse weight; Imalgene 1000, Merial, France) and then, euthanized by transcardial perfusion with 0.9% NaCl, followed by perfusion with 4% paraformaldehyde (PFA; Sigma-Aldrich). Brains were removed, fixed overnight with 4% PFA, and immersed in a 30% sucrose solution. Finally, tissues were cryopreserved, and 40-μm-thick coronal sections were obtained using a freezing cryostat-microtome (Leica CM 3050S, Leica Microsystems, Nussloch, Germany).

### Western blotting

Cortical tissue or cell cultures were lysed using RIPA lysis buffer (0.15 M NaCl, 0.05 M Tris, 5 mM ethylene glycol tetraacetic acid, 1% Triton X-100, 0.5% deoxycholic acid, 0.1% sodium dodecyl sulphate, and 10 mM dichlorodiphenyltrichloroethane), with a cocktail of proteinase inhibitors (Roche Diagnostics Ltd., Mannheim, Germany). Total protein concentration was determined using the BCA assay (Thermo Scientific). The samples were separated in an 8%, 10% or 12.5% acrylamide gel (Applichem, Darmstadt, Germany), by sodium dodecyl sulphate-polyacrylamide gel electrophoresis (Mini-PROTEAN^®^ Tetra Handcast, Bio-Rad, CA, USA), in a Tris-glycine running solution (pH 8.3; Acros Organics, Geel, Belgium) at room temperature and were transferred onto a polyvinylidene difluoride membrane (GE Healthcare, Little Chalfont, UK) for 60 or 120 min. Membranes were blocked with Tris-buffered saline containing 0.05% Tween 20 (TBST; Sigma) and 0.1% gelatin (Sigma) and incubated with the primary and secondary antibodies detailed in Additional file 2: Table S1. Protein expression was normalized with housekeeping targets. Proteins were detected by enhanced chemiluminescence exposure (Chemidoc™ MP imaging system (BioRad Laboratories, CA, USA) and levels were determined by densitometric analysis using the open-source image processing program ImageJ software (National Institutes of Health). Representative images of protein bands are shown in Additional file 3.

### Immunohistochemistry

The immunoassays were performed as described by us [24]. Briefly, brain slices were incubated in a blocking solution containing 2% of horse serum (Thermo Fisher Scientific) and 0.3% Triton X-100 (Thermo Fisher Scientific) diluted in 0.1 M PBS for 2 h at room temperature. Then, slices were incubated in the following primary antibodies diluted in the blocking solution for 72 h at 4 °C: rat anti-cluster of differentiation molecule 11b (CD11b) (1:500; AbD Serotec, Oxfordshire, UK), mouse anti-GFAP (1:500; BD Biosciences) or rabbit anti-Ago2 (1:100; Cell Signaling). After that, sections were rinsed in PBS and incubated with the respective secondary antibodies and Hoechst- 33342 (1:1000; Life Technologies) diluted in a solution containing 0.3% Triton X-100 in 0.1 M PBS for 2 h at room temperature: Alexa Fluor-488 donkey anti-rat or anti-mouse, and Alexa Fluor-594 donkey anti-rabbit (all 1:500; all Life Technologies). Finally, tissue sections were rinsed in PBS and mounted in Fluoroshield Mounting Medium (Abcam Plc., Cambridge, UK). Representative images were acquired using an AxioObserver LSM 710 confocal microscope (Carl Zeiss) under a 40× oil immersion objective and are provided in Additional file 4.

### Data analysis

**Fig. 2:**
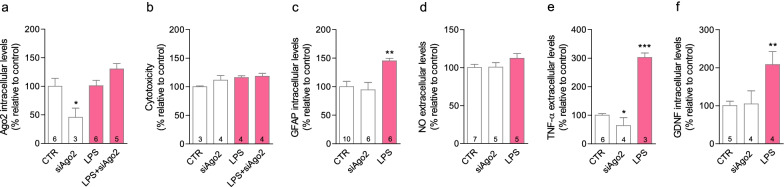
Ago2 is not involved in LPS-induced astrocyte activation. Direct exposure to lipopolysaccharide (LPS, 100 ng/ml) did not change Ago2 levels (**a**) or caused cytotoxicity in primary astrocytes (**b**). LPS-activated primary astrocytes overexpressed GFAP (**c**) and GDNF (**f**), measured by western blot, and released TNF-α (**e**), measured by ELISA. Ago2 silencing decreased Ago2 levels (**a**) and lowered the basal release of TNF-α (**e**). No effect was observed on NO (**d**). Data are expressed as the mean ± SEM of the indicated number of repeats and as a percentage relative to untreated controls (*p < 0.05, **p < 0.01, ****p < 0.0001 compared to untreated controls; one-way ANOVA for all figures; in **e**, Student’s *t* test was used for the comparison between CTR and siAgo2). *Ago2* argonaute-2, *LPS* lipopolysaccharide, *NO* nitric oxide, *TNF-α* tumor necrosis factor-alpha, *GDNF* glial-derived neurotrophic factor

**Fig. 3:**
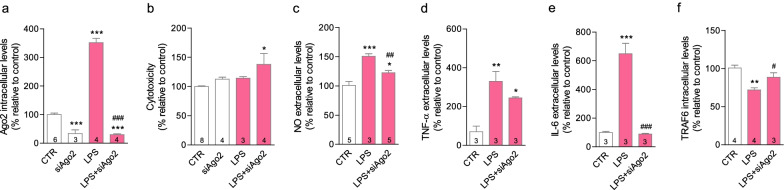
Ago2 silencing reduces microglial inflammatory responses. LPS (LPS, 100 ng/ml) increased Ago2 intracellular levels in primary microglia, while the transfection with 0.05 µM of Ago2 siRNA reduced the intracellular levels of Ago2, even after LPS stimulation, measured by western blotting (**a**). Ago2 silencing was cytotoxic only in conjunction with LPS (**b**). Ago2-silenced microglial revealed lower levels of NO (**c**) and IL-6 (**e**), but not TNF-α (**d**), in an inflammatory environment, measured by ELISA. TRAF6 expression was reduced after LPS challenge but Ago2 silencing counteracted this effect, measured by western blotting (**f**). Data are expressed as the mean ± SEM of the indicated number of repeats and as a percentage relative to untreated controls (*p < 0.05, **p < 0.01; ***p < 0.001, compared to untreated controls; ^#^*p* < 0.05, ^##^*p* < 0.01, ^###^*p* < 0.001, compared to LPS-activated cells; one-way ANOVA for all figures; in **d**, Student’s *t* test was used for the comparison between CTR and LPS). *Ago2* argonaute-2, *IL-6* interleukin-6, *LPS* lipopolysaccharide, *NO* nitric oxide, *TNF-α* tumor necrosis factor-alpha, *TRAF6* TNF receptor-associated factor 6

**Fig. 4:**
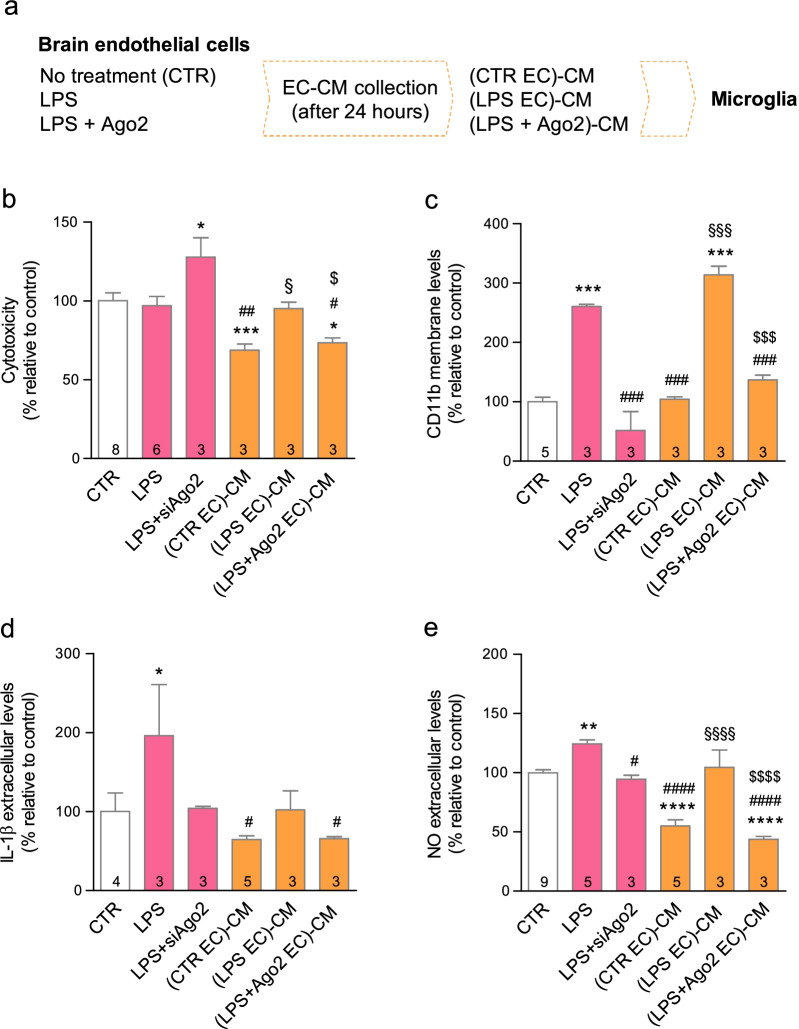
Ago2-restored endothelium normalizes microglia response. Schematic representation of the experimental setup (**a**). Endothelial cell-conditioned media (EC-CM) from cells exposed to LPS and treated with Ago2 (0.4 nM) ((LPS + Ago2 EC)-CM) reproduced the protective effect of EC-CM collected from healthy endothelial cells ((CTR EC)-CM), measured by LDH activity assay (**b**). Microglial cells exposed to (LPS + Ago2 EC)-CM presented CD11b levels similar to untreated cells (CTR) and cells exposed to (CTR EC)-CM, measured by western blotting, while (LPS EC)-CM had the same effect as the LPS treatment (**c**). EC-CM had no significant effect over IL-1β release (**d**), but (CTR EC)-CM and (LPS + Ago2 EC)-CM decreased NO below control levels (**e**). Data are expressed as the mean ± SEM of the indicated number of repeats and as a percentage relative to untreated controls (*p < 0.05, **p < 0.01, ***p < 0.001, ****p < 0.0001, compared to untreated controls; ^#^*p* < 0.05, ^##^*p* < 0.01, ^###^*p* < 0.001, ^####^*p* < 0.0001, compared to LPS-activated cells; ^§^*p* < 0.05, ^§§§^*p* < 0.001, ^§§§§^*p* < 0.0001, compared to (CTR EC)-CM; ^$^*p* < 0.05, ^$$$^*p* < 0.001, ^$$$$^*p* < 0.0001, compared to (LPS EC)-CM; one-way ANOVA; in **d**, Student’s *t* test was used for the comparison between CTR and LPS)). *Ago2* argonaute-2, *CD11b* cluster of differentiation molecule 11b, *EC-CM* endothelial cell-conditioned media, *IL-1β* interleukin-1 beta, *LPS* lipopolysaccharide, *NO* nitric oxide

**Fig. 5:**
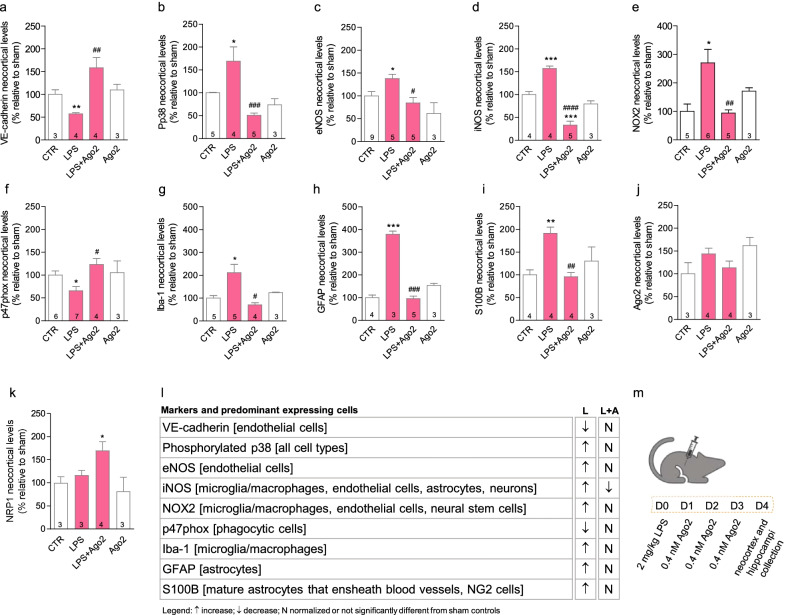
Systemic administration of Ago2 restores endothelial barrier function and normalizes glial activation in the cortex. Mice injected intraperitoneally with lipopolysaccharide (LPS, 2 mg/kg) showed the activation of p38 signaling pathway, i.e., increased p38 phosphorylation (Pp38) (**b**) and produced higher levels of eNOS (**c**), iNOS (**d**), NOX2 (**e**), Iba-1 (**g**), GFAP (**h**) and S100B (**i**), and lower levels of VE-cadherin (**a**) and p47phox (**f**). LPS-injected mice treated with Ago2 (0.4 nmol/l) had marker levels similar to sham controls (animals injected with phosphate-buffered saline, CTR), with the exception of the Pp38 and iNOS markers. The same pattern of reduction below control levels was obtained with Ago2 injection in sham controls. Ago2 cortical levels did not change significantly in any of the experimental conditions (**j**). The levels of NRP1 were only raised in LPS-injected mice treated with Ago2 (**k**). The table summarizes data from these experiments (**l**). Schematic representation of the experimental setup used herein (and in this figure) (**m**). Data are expressed as the mean ± SEM of the indicated number of repeats and as a percentage relative to sham controls (*p < 0.05, **p < 0.01, ***p < 0.001, ****p < 0.0001 compared to sham controls; ^#^*p* < 0.05, ^##^*p* < 0.01, ^####^*p* < 0.0001 compared to LPS-injected animals; one-way ANOVA; in **a**, **c** and **f**, Student’s *t* test was used for the comparison between CTR and LPS). *Ago2* argonaute-2, *eNOS* endothelial nitric oxide synthase, *GFAP* glial fibrillary acidic protein, *Iba-1* ionized calcium-binding adaptor molecule-1, *iNOS* inducible nitric oxide synthase, *LPS* lipopolysaccharide, *NG2* oligodendrocyte precursor cells, *NO* nitric oxide, *NOX2* NADPH oxidase 2, *NRP1* neuroplilin-1, *p47phox* neutrophil cytosol factor 1, *Pp38* phosphorylated p38 mitogen-activated protein kinases signaling pathway, *S100B* S100 calcium-binding protein B, *VE-cadherin* vascular endothelial-cadherin

**Fig. 6:**
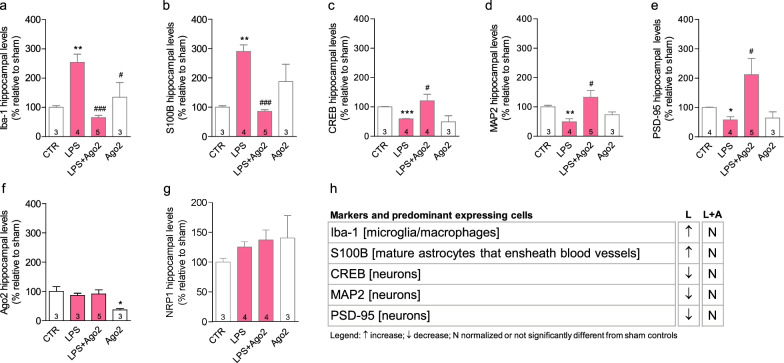
Systemic administration of Ago2 normalizes inflammatory activation and induces neuroprotection in the hippocampus. LPS-injected mice (LPS, 2 mg/kg) produced higher levels of Iba-1 (**a**) and S100B (**b**), and lower levels of CREB (**c**), MAP2 (**d**) and PSD-95 (**e**). Mice treated with Ago2 (0.4 nmol/L) had marker levels similar to sham controls (animals injected with phosphate-buffered saline, CTR), with the exception of the Iba-1 marker. Similar to the mouse cortex, the Ago2 hippocampus levels did not change significantly in the experimental conditions, with the exception of the Ago2-injected mice, in which a decrease of Ago2 levels occurred (**f**). NRP1 levels did not change significantly in any of the experimental conditions (**g**). The table summarizes data from these experiments (**h**). Data are expressed as the mean ± SEM of the indicated number of repeats and as a percentage relative to sham controls (*p < 0.05, **p < 0.01, ***p < 0.001, ****p < 0.0001 compared to sham controls; ^#^*p* < 0.05, ^###^*p* < 0.001, ^####^*p* < 0.0001 compared to LPS-injected animals; one-way ANOVA; in **c** to **e**, Student’s *t* test was used for the comparison between CTR and LPS). *Ago2* argonaute-2, *CREB* cAMP response element-binding protein, *Iba-1* ionized calcium-binding adaptor molecule-1, *LPS* lipopolysaccharide, *MAP2* microtubule-associated protein, *NRP1* neuropilin-1, *PSD-95* postsynaptic density protein-95, *S100B* S100 calcium-binding protein B

Statistical analysis was performed using GraphPad Prism 8.1 (GraphPad Software, CA, USA). Statistical significance was determined using one-way ANOVA, followed by Dunnett’s post-comparisons test and Bonferroni’s post-comparisons test, in all figures; we performed Student’s *t* test in Figs. 1i, 3d, 4d, 5a, c, f, 6c–e, to compare CTR versus LPS and in Fig. 2e, to compare CTR versus siAgo2. Statistical significance was considered relevant for *p* values < 0.05. Data are expressed as mean ± standard error of mean, determined from at least three independent experiments, performed in duplicate, except for western blotting experiments (no duplicates).

## Results

### LPS induces Ago2 downregulation and endothelial activation

Primary brain endothelial cells were exposed to LPS (100 ng/ml) to mimic contact with an inflammatory environment [26–30]. These cells express Ago2 and its receptor, neuropilin 1 (NRP1) [19] (Additional file 1). Exposure to LPS significantly downregulated Ago2 intracellular levels (LPS = 41.9 ± 7.5%, **p < 0.01), similarly to the effect observed with Ago2 silencing (siAgo2 = 35.7 ± 3.5%, **p < 0.01) (Fig. 1a). We have shown that a concentration of 50 ng/ml siRNA knockdowns intracellular Ago2 [18]. Ago2 silencing compromised cell survival (siAgo2 = 64.8 ± 4.9%, *p < 0.05) (Fig. 1b) and induced necrosis or late apoptosis, as suggested by the release of LDH through the disrupted plasma membrane (LPS = 170.5 ± 3.2%, ****p < 0.0001) (Fig. 1c). We measured the extracellular levels of Ago2 by ELISA, but values were below the detection range and sensitivity threshold value. Thus, we considered that Ago2 was not released. LPS had no effect on cell viability nor caused toxicity (Fig. 1b, c), but significantly decreased total protein content (Fig. 1d). The application of Ago2 alone did not change Ago2 intracellular levels (Ago2 = 72.3 ± 9.2%) (Fig. 1a) nor affected cell viability, toxicity, or protein content (Fig. 1b–d); however, it increased NRP1 (Ago2 = 148.5 ± 6.4%, ***p < 0.001) (Fig. 1e). In contrast, Ago2 silencing did not change the expression levels of NRP1 (siAgo2 = 105.9 ± 7.6%), while LPS exposure significantly decreased the expression levels of NRP1 (LPS = 21.5 ± 9.2%, ***p < 0.001) (Fig. 1e). To validate Ago2 treatment, we confirmed that cells exposed to LPS and Ago2 maintained NRP1 expression similar to control cells (LPS + Ago2 = 89.3 ± 11.3%). Conversely, intracellular Ago2 levels were increased upon Ago2 exogenous co-application (0.4 nM) (LPS + Ago2 = 138.5 ± 15.8%, ^####^*p* < 0.0001) (Fig. 1f). This concentration ensures maximum miRNA delivery to primary brain endothelial cells [18], to secure RNA-induced silencing complex (RISC) activity. Then, we evaluated the contribution of a protein-degrading mechanism, autophagy, by treating cells with a late-stage inhibitor of this process, bafilomycin A1 (BafA1). The treatment with BafA1 for 3 h increased intracellular Ago2 (LPS + BafA1 = 138.5 ± 13.9%, ^####^*p* < 0.0001) (Fig. 1f). We tested other periods of incubation with BafA1, albeit with no effect on Ago2 levels (data not shown). Using the same experimental conditions, we measured the release of nitric oxide (NO) (Fig. 1g). NO is a vasoregulator that inhibits platelet aggregation and leukocyte adhesion [19]. While LPS increased NO levels (LPS = 186.5 ± 15.7%, ***p < 0.001), as expected [4], Ago2 treatment inhibited NO release (LPS + Ago2 = 41.7 ± 15.0%, ^####^*p* < 0.0001). Autophagy inhibition failed to change the NO response to LPS (LPS + BafA1 = 219.5 ± 32.8%, ***p < 0.001) (Fig. 1g). LPS downregulated the expression of VE-cadherin (LPS = 63.5 ± 22.5%, *p* = 0.0307), which is responsible for the assembly of adherens junctions and BBB integrity [31]. Ago2 treatment recuperated VE-cadherin expression (LPS + Ago2 = 134.6 ± 22.5%, ^#^*p* < 0.05) (Fig. 1h) and the inhibition of autophagy resulted in VE-cadherin levels similar to untreated cells, as well (LPS + BafA1 = 100.1 ± 22.7%) (Fig. 1h). LPS administration enhanced the release of cytokines involved in angiogenesis, TNF-α (LPS = 225.0 ± 28.9%, *p < 0.05) and IL-6 (LPS = 319.9 ± 37.7%, ****p < 0.0001), as expected [32, 33], but Ago2 treatment failed to produce a significant effect (Fig. 1i, j, respectively). In sum, the loss of Ago2 appears to be correlated with specific markers of endothelial activation.

### Microglial, but not astrocytic Ago2, is necessary for LPS-induced cell activation

Astrocytes are physically associated with, and significantly contribute to regulate blood vessel function [34]. Contrary to brain endothelial cells, primary astrocytes did not reveal significant differences in Ago2 expression after LPS exposure. While Ago2 silencing per se reduced Ago2 levels (siAgo2 = 45.7 ± 16.1%, *p < 0.05), Ago2 expression was maintained and slightly increased following LPS stimulation (LPS + siAgo2 = 130.3 ± 9.4%) (Fig. 2a), and reproduced the same results as LPS alone in the following panels. The astrocytic population did not demonstrate significant changes in cell viability (Fig. 2b). Astrocytes were activated by LPS exposure, as observed by increased expression of glial fibrillary acidic protein (GFAP) (LPS = 145.2 ± 4.7%, **p < 0.01) (Fig. 2c), TNF-α release (LPS = 302.9 ± 15.5%, ****p < 0.001) (Fig. 2e) and expression of glial cell-derived neurotrophic factor (GDNF) (LPS = 208.3 ± 34.2%, *p < 0.05) (Fig. 2f). However, we did not register any changes in NO levels (Fig. 2d). Ago2 silencing alone only resulted in a significant decrease regarding the basal release of TNF-α (siAgo2 = 64.0 ± 14.0%, *p* = 0.0271) (Fig. 2e). Microglia participate in the inflammatory response and contribute to the maintenance of BBB integrity [3]. Contrary to brain endothelial cells, under inflammatory conditions, microglia overexpressed Ago2 (LPS = 352.0 ± 15.2%, ***p < 0.001) (Fig. 3a), with no impact on cell viability (Fig. 3b). In microglia, Ago2 silencing alone (0.05 µM) lowered Ago2 intracellular levels (siAgo2 = 34.0 ± 12.7%, ***p < 0.001) as well as in the presence of LPS (LPS + siAgo2 = 30.0 ± 2.7%, ***p < 0.001) (Fig. 3a), highlighting important differences with astrocytes. Only in the latter condition, cell toxicity was induced (LPS + siAgo2 = 137.9 ± 18.5%, *p < 0.05) (Fig. 3b). However, in these conditions, Ago2 silencing lowered the release of NO (LPS + siAgo2 = 122.4 ± 3.9%, ^##^*p* < 0.01 (Fig. 3c) and normalized IL-6 (LPS + siAgo2 = 88.4 ± 5.5%, ^###^*p* < 0.001) (Fig. 3e). The opposite effect was obtained regarding the expression of TNF receptor-associated factor 6 (TRAF6) (LPS + siAgo2 = 88.5 ± 5.9%, ^#^*p* < 0.05) (Fig. 3f) versus the decrease induced after LPS stimulation (LPS = 71.9 ± 2.9%, **p < 0.01). TRAF6 is a regulator of the immune response [35]. No changes were produced by Ago2 silencing on LPS-stimulated TNF-α release (Fig. 3d).

### Ago2-restored endothelium reduces microglia activation

The endothelial secretome shapes microglia responses and endothelial-activated microglial cells adopt a neurotoxic profile [36]. We exposed primary microglia to the conditioned media of primary brain endothelial cells (EC-CM) previously stimulated with LPS alone (100 ng/ml) ((LPS EC)-CM) or treated with Ago2 (0.4 nM) ((LPS + Ago2 EC)-CM), or left untreated (CTR EC)-CM) (Fig. 4a). (CTR EC)-CM significantly reduced microglial basal cell death, but this effect was lost with (LPS EC)-CM ((CTR EC)-CM = 68.7 ± 3.9%, ***p < 0.001); (LPS EC)-CM = 95.0 ± 4.0%, ^§^*p* < 0.05)). Ago2 treatment retained the protective quality of (CTR EC)-CM ((LPS + Ago2 EC)-CM = 73.5 ± 3.1%, *p < 0.05; ^$^*p* < 0.05) (Fig. 4b). Microglia exposed to (LPS + Ago2 EC)-CM presented CD11b levels similar to cells either left untreated or exposed to (CTR EC)-CM, and significantly different from cells exposed to (LPS EC)-CM, suggesting a strong attempt to prevent cell activation ((CTR EC)-CM = 104.1 ± 4.2%; (LPS EC)-CM = 313.9 ± 14.6%, ***p < 0.001, ^§§^*p* < 0.01; (LPS + Ago2 EC)-CM = 136.7 ± 8.4%, ^$$$^*p* < 0.05)) (Fig. 4c). Ago2 silencing in the presence of LPS also prevented CD11b overexpression (LPS + siAgo2 = 51.6 ± 32.1%, ^###^*p* < 0.001), although this condition raised cell death (LPS + siAgo2 = 127.7 ± 12.2%, *p < 0.05 (Fig. 4b). Lastly, IL-1β levels were not significant changed by any of the EC-CM, although cells were responsive to LPS stimulation (LPS = 196.1 ± 64.6%, *p = 0.0484) (Fig. 4d). On the other hand, (CTR EC)-CM and (LPS + Ago2 EC)-CM efficiently reduced NO below the levels obtained by untreated cells or cells exposed to (LPS EC)-CM ((CTR EC)-CM = 55.2 ± 5.2%, ****p < 0.0001; (LPS EC)-CM = 104.5 ± 14.6%, ^§§§§^*p* < 0.0001; (LPS + Ago2 EC)-CM = 43.8 ± 2.4%, ^$$$$^*p* < 0.0001) (Fig. 4e). Hence, the secretome of either Ago2-restored endothelium or normal brain endothelial cells shared the same properties.

### Systemic administration of Ago2 normalizes endothelial and glial activation in the mouse cortex

Mice were injected intraperitoneally with LPS (2 mg/kg) to induce BBB disruption and brain tissue inflammation [24, 36]. Then, mice received daily intraperitoneal injections of Ago2 (0.4 nM), as published by us and others [18, 25] (Fig. 5m) and we evaluated markers associated to LPS activation of endothelial and glial cells, namely VE-cadherin, phosphorylated p38 (Pp38), eNOS, iNOS, NOX2, p47phox, Iba-1, GFAP and S100B (Fig. 5a–j). As expected, all markers with the exception of VE-cadherin and p47phox were increased upon LPS administration (LPS = 57.4 ± 2.3%, **p = 0.0030 (VE-cadherin); LPS = 169.0 ± 31.3%, *p < 0.05 (Pp38); LPS = 137.7 ± 9.6%, *p = 0.0117 (eNOS); LPS = 157.2 ± 5.3%, ***p < 0.001 (iNOS); LPS = 270.2 ± 47.3%, *p < 0.05 (NOX 2); LPS = 65.6 ± 9.2%, *p = 0.0113 (p47phox); LPS = 211.5 ± 36.4%, *p = 0.05 (Iba-1); LPS = 378.6 ± 14.5%, ***p < 0.001 (GFAP); LPS = 191.3 ± 13.5%, **p < 0.01 (S100B)). No changes were produced on NRP1 levels (LPS = 116.7 ± 10.4%). Ago2 treatment (0.4 nM) normalized the expression of all markers with the exception of iNOS, which was decreased below control levels, and NRP1, which was upregulated (LPS + Ago2 = 109.9 ± 12.6%, ^##^*p* < 0.01 (VE-cadherin); LPS + Ago2 = 50.8 ± 4.9%, ^###^*p* < 0.001 (Pp38); LPS + Ago2 = 84.6 ± 11.8%, ^#^*p* < 0.05 (eNOS); LPS + Ago2 = 33.2 ± 8.7%, ***p < 0.001 (iNOS); LPS + Ago2 = 94.33 ± 11.1%, ^##^*p* < 0.01 (NOX2); LPS + Ago2 = 123.4 ± 13.2%, ^#^*p* < 0.05 (p47phox); LPS + Ago2 = 70.7 ± 9.0%, ^#^*p* < 0.05 (Iba-1); LPS + Ago2 = 95.4 ± 11.4%, ^###^*p* < 0.001 (GFAP); LPS + Ago2 = 95.9 ± 9.3%, ^##^*p* < 0.01 (S100B); (LPS + Ago2 = 170.3 ± 18.9%, *p < 0.05)). Ago2 injection per se did not produce any significant changes in these markers or over Ago2 tissue levels (Fig. 5j). These data, summarized in Fig. 5m, support the hypothesis of an anti-inflammatory effect produced by Ago2 via the deactivation of inflamed brain endothelium.

### Systemic administration of Ago2 normalizes inflammatory response and induces neuroprotection in the mouse hippocampus

We measured the expression of microglial marker Iba-1 and astrocyte marker S100B, which identifies astrocytes in direct contact with blood vessels, to evaluate whether Ago2 could reverse the glial and neuronal LPS-induced damage in the hippocampus [37]. As expected, glial markers were increased upon LPS administration [LPS = 254.3 ± 27.9% (Iba-1); LPS = 290.2 ± 22.5% (S100B); **p < 0.01 (Fig. 6a, b)]. Ago2 treatment (0.4 nM) restored the expression of Iba-1 (LPS + Ago2 = 64.8 ± 7.5%, ^###^*p* < 0.001) and S100B (LPS + Ago2 = 85.8 ± 5.7%, ^####^*p* < 0.0001). No changes were produced on NRP1 levels (LPS = 125.4 ± 9.2%; LPS + Ago2 = 137.6 ± 16.8%). To disclose if the normalized inflammatory response was accompanied by an effect over neuronal function and synaptic activity, we evaluated the expression of CREB, MAP2 and PSD-95 [37–39]. Herein, as expected, we observed a reduction in the expression of CREB, MAP2 and PSD-95 upon LPS administration [LPS = 59.0 ± 1.2%, ***p < 0.001 (CREB); LPS = 49.2 ± 10.3%, **p = 0057 (MAP2); LPS = 58.1 ± 10.7%, *p = 0.0109 (PSD-95) (Fig. 6c–e)]. Ago2 treatment normalized the expression of these markers (LPS + Ago2 = 120.5 ± 22.8% (CREB); LPS + Ago2 = 132.6 ± 23.8% (MAP2); LPS + Ago2 = 211.9 ± 56.0% (PSD-95); ^#^*p* < 0.05. Ago2 per se did not have an effect (Fig. 6a–e). Finally, the Ago2 hippocampus levels did not change in the experimental conditions, except for the Ago2-injected mice, in which a decrease was observed (Ago2 = 36.8 ± 4.5%, *p < 0.05) (Fig. 6f). The table summarized data that support the hypothesis that Ago2 may positively impact neuronal activity upon inflammatory challenge (Fig. 6h).

## Discussion

The role of the brain endothelium in inflammation-induced barrier loss and parenchymal damage remains poorly explored, despite being a key target for intravascular therapies addressing injury in the central nervous system [37]. The first report exploring Ago2 in vascular function showed that silencing this protein alone inhibited proliferation, enhanced apoptosis and suppressed the formation of tubules of HUVEC [16]. Thus, our first aim was to clarify the role of Ago2 in brain endothelial cells and identify changes in function, via Ago2, when exposed to an inflammatory cue, i.e., LPS. LPS stimulation alters the expression of junctional proteins and triggers the production of several inflammatory mediators (in vitro models), and disrupts the BBB (in vivo models) [2]. Our data showed that LPS-activated brain endothelial cells expressed lower Ago2 levels, an effect reproduced with Ago2 silencing. However, only the latter specifically compromised cell survival, in accordance with the work of Asai and colleagues [16]. The exogenous application of Ago2 increased intracellular levels, likely via NRP1-mediated internalization. NRP1 is the main Ago2 receptor (whether Ago2 is carrying miRNA or not) although it can also bind to multiple ligand families such as class III semaphorins and the VEGF family [20]. Neuronal, endothelial, and epithelial cells are the main cell types expressing NRP1 and are thus responsive to extracellular Ago2 [40], which is particularly relevant for understanding the outcomes of our in vivo model. Interestingly, LPS stimulation downregulated NRP1, an effect that was shown by others to provide positive signals for cell survival (HUVEC) through the regulation of growth factor receptors [41]. The reduction of NRP1 expression could be a consequence of the protein-degrading mechanism autophagy, which occurs in the inflamed endothelium [42], possibly to maintain cell viability. In primary macrophages, LPS (100 ng/ml for 12 h) also mediated NRP1 suppression through the activation of TLR4–NF-κB p50 and p65 pathways. However, this result is not consistently observed in all cells exposed to LPS (e.g., mouse embryonic fibroblasts, rat fibroblasts, HUVEC, and human vascular smooth muscle cells), suggesting a difference in the regulation of NRP1 depending on cell type, cell location, cell immortalization and their different response and/or sensibilization to LPS administration [43]. In contrast, NRP1 expression was maintained with Ago2 silencing, as a likely compensatory mechanism to maintain endothelial function through VEGF interactions [44]. The exogenous application and subsequent internalization of Ago2, in the presence of LPS, may imply other forms of Ago2 internalization, such as vesicular structures [45] or a different neuropilin receptor. However, our data show that Ago2 and LPS co-administration replenished NRP1 expression, allowing for Ago2 proper internalization. Bae and colleagues also showed that, although hypoxia and nutrient deprivation decreased NRP1 expression, it did not change NRP2 levels [41]. Ago2 alone upregulated NRP1; this is possibly a response to the rise in available ligand, i.e., exogenous Ago2 (which is not naturally present in cell culture medium) as a cue to promote internalization. However, healthy cells did not internalize Ago2, or alternatively, were able to degrade it, maintaining their intracellular levels constant. Since Ago2 was not released, we hypothesized that the LPS-induced decrease of intracellular Ago2 occurred by autophagy [46]. Accordingly, the inhibition of autophagy in the presence of LPS increased Ago2 levels similarly to Ago2 supplementation. Changes in Ago2 levels influence miRNA availability and delivery, and autophagy-mediated degradation of Ago2 during LPS challenge could decrease miRNA processing and facilitate the translation of inflammatory targets. Contrary to Ago2 treatment (maintained over a period of 24 h), the inhibition of autophagy, which also increased intracellular Ago2 (within 3 h), did not reduce LPS-induced NO release. Exogenously administered Ago2 possibly maintains the processing of Ago2–miRNA complexes in the cell cytoplasm [8] for a longer period of time, shutting translation off for targets involved in the NO signaling pathway. Recently, NO release from endothelial cells was found considerably decreased when miR-195 and miR-582 were up-regulated [47]. The target of these miRNA is eNOS and we showed that Ago2 reduced eNOS and iNOS expression in the LPS-injected mice, further supporting a modulatory role of Ago2 in this pathway. Thus, our results revealed that, under inflammatory conditions, Ago2 can be considered, indirectly, a pro-angiogenic factor since low NO levels are deemed pro-angiogenic while high amounts are anti-angiogenic. Since excessive NO levels also correlate with higher VE-cadherin complex disruption in murine microvascular endothelial cells [48], Ago2-induced reduction of NO could have contributed to normalize VE-cadherin levels. The inhibition of autophagy recuperated the loss of VE-cadherin caused by LPS. LPS triggers a series of events leading to tyrosine phosphorylation of VE-cadherin, its disassembly from the adherens junction, and lysosomal degradation [49]. Others have shown that a different autophagy inhibitor (3-methyladenine) prevented the cleavage and consequent loss of VE-cadherin at adherens junctions, in a model of acute lung injury [50]. Ago2 treatment did not affect the release of TNF-α, or IL-6 induced by LPS in brain endothelial cells. Most authors agree on a pro-angiogenic effect in vivo and an anti-angiogenic role in vitro for TNF-α. However, Sainson and colleagues described a temporal effect: TNF-α initially blocks signaling through VEGFR2, but once inflammation is resolved, it induces a tip cell phenotype [32]. Ago2 did not appear to regulate TNF-α, but the maintenance of high levels of TNF-α during LPS stimulation and Ago2 co-treatment suggests a pro-angiogenic purpose. Lowering the release of IL-6 could be beneficial to vascular activity since high expression can promote defective angiogenesis [33], but no significant reduction of IL-6 release induced by Ago2 was observed.

Then, we assessed the role of Ago2 in primary astrocytes. These are crucial regulators of central nervous system homeostasis and BBB function [51]. In vitro, the full range of astrocytic activation can be limited in pure cultures; the presence of contaminating microglia enhances the response to LPS [52], which may account for the tamer response regarding NO release. Moreover, LPS did not change Ago2 levels and was able to overcome Ago2 silencing, suggesting a compensatory mechanism. LPS-activated astrocytes exhibited GDNF overexpression and higher TNF-α release. Exogenous TNF-α can induce the astrocytic expression and secretion of GDNF in vitro and vivo, while the disruption of TNFR1 signaling cancels this effect. Accordingly, Ago2 silencing lowered the basal release of TNF-α and maintained GDNF levels similar to control, as described in vitro and in vivo by Brambilla and colleagues [53]. Thus, astrocytic Ago2 does not appear to play a role in LPS-induced inflammation.

We then characterized the role of Ago2 in primary microglial cultures stimulated with LPS. Contrary to brain endothelial cells, intracellular Ago2 levels were upregulated in activated microglia. Microglial Ago2 does not seem relevant for cell survival since silencing did not produce significant cell death (only in combination with LPS stimulation). Ago2 accumulation was accompanied by higher levels of IL-6, TNF-α and NO. The effect of LPS on Ago2 has been addressed on peripheral macrophages [54], which share functional similarities with microglia. In that study, the early response to LPS caused Ago2 phosphorylation and miRNA dissociation, thus shutting down miRNA-mediated repression and enhancing cytokine synthesis. Authors then showed that a prolonged exposure to LPS reversed the process. In their work, no changes in Ago2 intracellular levels or localization were reported; moreover, Ago2 was found to be the most abundant argonaute protein in their model (RAW 264.7 cells). Ago2 is commonly perceived as the most relevant family member given its slicer activity and its higher relative abundance in the cell. In our work, Ago2 silencing did not change TNF-α levels but reduced LPS-induced release of IL-6 and NO, suggesting an important and specific role of Ago2 in these pathways. Others have reported that the four Ago proteins can function redundantly and, upon Ago2 downregulation, Ago1 and Ago3 can become functional substitutes in vitro [27, 55, 56], maintaining to some degree RISC activity and translation off. Accordingly, we found that TRAF6 levels were similar to control cells upon Ago2 silencing. TRAF6 downregulation occurs after prolonged LPS stimulation to avoid excessive immune response [35, 57]. Since the release of IL-6 and NO was prevented, this mechanism could have been delayed to some extent. Moreover, if RISC is compromised because of Ago2 silencing, miR-146a-, or miR14b-, or miR-124-dependent degradation of TRAF6 may not occur [58], which should have led to sustained inflammatory release in the presence of LPS (it only occurred for TNF-α). These observations further fuel the idea that the role of Ago2 is complex, and it modulates cell responses differently.

To clarify the process of endothelial regulation, via Ago2, we evaluated microglial activation induced by endothelial cell-conditioned media. Brain endothelial cells are a trophic vault for factors that regulate immune responses and parenchymal regeneration [3]. While media from healthy endothelial cells reduced basal cell death by likely enhancing survival/proliferation or slowing down cell death processes [59], media obtained from LPS-stimulated endothelial cells abolished this effect. Conversely, Ago2 treatment modulated this pool of factors, secreted by endothelial cells, increasing again cell survival and favoring a surveying microglial state. While we did not observe a significant impact on the release of microglial IL-1β, conditioned media from healthy or Ago2-treated activated brain endothelial cells greatly reduced NO below control levels. If the microenvironment favors proliferation, microglia lower NO, by decreasing iNOS expression and protein kinase G signaling, which results in increased cell division [60]. Lastly, we used a recombinant protein to assess the specific role of Ago2 in the endothelial secretome. Ago2 may be acting alone or in a ribonucleoprotein complex with miRNA that are secreted by cells and/or that are already present in cell media. Recently, the content of small non-coding RNA contaminants was evaluated in fetal bovine serum (FBS), vesicle-depleted FBS and serum-free media. None were found to be free of small non-coding RNA contaminants [61]. To add complexity to the matter, the positive outcomes observed in the in vivo studies can also be a result of Ago2 possibly being delivered in vesicular structures that can transport lipids and other proteins that could hypothetically contribute to the observed results on endothelial cells, and consequently on the composition of their secretome [62]. Above all, the conditioned media from both healthy and activated brain endothelial cells treated with Ago2 had similar properties, and both elicited different responses than the media from activated endothelial cells.

A systemic challenge with 2 mg/kg LPS disrupts the BBB and results in significant glial activation [2, 24], which is best assessed by examining both pro- and anti-inflammatory markers [63]. Since LPS minimally penetrates the murine brain (about 0.025%) and BBB disruption does not enhance LPS uptake [64], this model induces a local effect on blood vessels (reproducing the in vitro data obtained with brain endothelial cells). It is also unlikely that Ago2 crosses the BBB, given its high molecular size (nearly 100 kDa). In light of our in vitro data, Ago2 is likely internalized by the brain endothelial cells that constitute the blood–brain barrier (BBB), via NRP1, reestablishing vascular function, with consequent normalizing actions on neuronal and glial cells. In addition, our in vivo data indicated that there was no direct correlation between changes in Ago2 measured in the tissue lysates and the injection of Ago2 (alone or after LPS). In the neocortex, Ago2 levels remained unchanged and, in the hippocampus, Ago2 was either similar to sham animal levels or reduced. Considering the in vitro data, we first confirmed that LPS lowered VE-cadherin expression and then focused on a signaling pathway responsive to stress stimuli (p38 mitogen-activated protein kinases), enzymes that are involved in NO synthesis (eNOS and iNOS), oxidative stress (NOX2 and the cytosolic Nox2 organizer p47phox), and markers for glial activation (Iba-1, GFAP and S100B) [65, 66]. The systemic administration of Ago2 normalized targets associated with vascular function and immune cell activity, and decreased iNOS levels, possibly because of its broader cellular expression (or Ago2 specificity on this pathway). Along the same lines as our in vitro data, others have identified miR-939 and miR-26a as blockers of iNOS protein synthesis [55]. By making Ago2 available again, Ago2-miRNA complexes can reassemble and perform their function. Recently, Carbonell and Gomes extensively reviewed the relationship between cellular redox status and miRNA expression, listing several molecules that inhibit NOX2 expression, such as miR-448-3p, miR-124-5p and miR-652 [67]. Finally, miR-451 levels depend on the maintenance of Ago2 levels. Yet, Ago2 expression was found to be reduced in p47phox-deficient macrophages, impairing miR-451 processing (data obtained in near anoxic conditions) [68]. In a macrophage cell line, LPS (100 ng/ml; the same concentration used by us in vitro) also induced a time-dependent decrease in p47phox expression (69), thus possibly reducing Ago2 expression. Indeed, few studies have focused on the role of Ago2 in inflammation-induced brain injury. A study conducted on peripheral blood mononuclear cells from post-traumatic stress disorder patients concluded that the chronic inflammation observed in these individuals could be triggered by downregulation of Ago2 and Dicer1, which impairs the generation of mature miRNA [70]. The miR-132-mediated Ago2 suppression in human dermal lymphatic endothelial cells affected the levels of miR-221 and miR146a, which are involved in angiogenesis and inflammation, respectively [56]. Hence, certain Ago2-miRNA complexes operate as key regulators of the inflammatory process [71]. In other contexts, miR-145-5p induces post-transcriptionally Ago2 expression, dictating miR-145-5p tumor suppressor activity (breast carcinoma cell lines) [72]. Conversely, the impairment of Ago2/miR-185-3p axis may promote colorectal cancer metastasis in colorectal cancer tissues [73]. In sum, Ago2 expression and/or effects may be conditioned by different miR, which are in turn expressed differently in terms of cell type and their response to a stimulus. It would be interesting to explore in the future which set of miRNA preferably binds to Ago2 and are internalized by brain endothelial cells, under inflammatory conditions, to assess if there are specific molecules responsible for the observed effects. Ago2 normalized the expression of GFAP and S100B. Elevated levels of S100B and GFAP are considered markers for astrocytic damage or dysfunction but S100B could be a more interesting target during the angiogenic process since it identifies astrocytes that ensheath blood vessels [74]. S100B can also be secreted and exhibit a cytokine-like activity, coordinating glia–glia and glia–neuron interactions [75]. Donato and colleagues postulated that these effects were achieved by the contact between S100B and the receptor for advanced glycation end products (RAGE), a multiligand receptor that propagates inflammatory stimuli and affects several neurotrophic and neurotoxic factors in inflammatory disorders. LPS injection slightly increased Ago2 detection in the neocortex, albeit not significantly. Microglia, which overexpress Ago2 in vitro and in vivo (Additional file 4) are likely contributing to this effect; there is also a higher microglial density in this brain region than in the hippocampus [76]. Ago2 overexpression is found in cancerous tissue and cell lines [17]. In this context, conflicting reports claim that Ago2 upregulation does not exert any effect on cell proliferation or migration [77], has an inhibitory effect on migration [78], or promotes invasiveness [79, 80]. LPS administration induces hippocampal microglia reactivity, which in turn secrete cytokines that may induce astrocytic activation [81]. Herein, these effects were normalized after the systemic injection of Ago2, similarly to what we had observed in the neocortex. LPS-induced systemic inflammation negatively affects cognitive function [82, 83], and our results demonstrated a significant decrease in the expression of CREB, MAP2 and PSD-95. CREB is critical for axonal outgrowth, synaptic plasticity, and memory formation [38]. MAP2 is involved in synaptic plasticity and neuronal cell death [37]. PSD-95 influences synaptic transmission and plasticity, learning and memory [39]. Ago2 injection caused the normal expression of these markers as a likely consequence of the previously described normalizing actions on glial cells. The Ago2 injection per se may have resulted in lower Ago2 than sham animals, possibly via a mechanism of negative regulation to counteract the trend towards elevated NRP1 expression in the hippocampus and stabilize signaling. Batassa and colleagues have reported that Ago2 silencing in the mouse hippocampus can alter RISC activity, which can affect learning and memory processes in these animals [84].

**Fig. 7:**
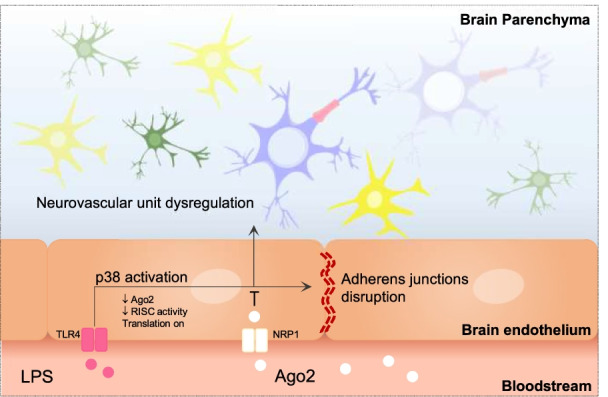
Proposed model for Ago2 regulation of the endothelial and glial crosstalk. LPS activation of the p38 signaling pathway promotes VE-cadherin downregulation and the transcription of mRNA related to pro-inflammatory mediators (e.g., cytokines). The translation of these transcripts is facilitated by low Ago2 levels and low RISC activity. These events trigger the activation of microglia and astrocytes associated with blood vessels, and cause neuronal damage. The exogenous application and internalization of Ago2, via NRP1, recuperates RISC activity, which is conducive to eNOS degradation and low NO, reducing glial activation and protecting neuronal cells. *Ago2* argonaute-2, *IL-6* interleukin-6, *LPS* lipopolysaccharide, *mRNA* messenger ribonucleic acid, *NO* nitric oxide, *NRP1* neuropilin-1, *p38* p38 mitogen-activated protein kinases signaling pathway, *RISC* RNA-induced silencing complex, *TNF-α* tumor necrosis factor alpha, *TLR4* toll-like receptor 4, *VE-cadherin* vascular endothelial-cadherin

Considering these results, we propose that Ago2 regulation of the neurovascular unit occurs as depicted in Fig. 7. LPS activation of the p38 signaling pathway in brain endothelial cells promotes the transcription of mRNA related to pro-inflammatory mediators and the disruption of the VE-cadherin complex. The translation of these transcripts (e.g., IL-6, TNF-α) is facilitated by low Ago2 levels and triggers glial activation. The exogenous application and internalization of Ago2, via NRP1, reduces eNOS expression and NO levels, restoring barrier integrity, and granting glial and neuronal protection.

## Conclusions

Ago2 is considered a rate-limiting factor in the miRNA processing pathway and the main miRNA transporter in the bloodstream, emphasizing the importance of both Ago2 intracellular and extracellular levels for adequate cellular responses. Herein, we describe how an inflammatory cue changed Ago2 levels differently in endothelial and glial cells, and how the exogenous application of Ago2 normalized endothelial function and Ago2 silencing prevented microglial activation. In vivo data recapitulated in vitro results showing that normalizing endothelial activation, via Ago2, attenuated or reestablished glial and neuronal activity, even after LPS injection. Considering the relevant role of the brain endothelium in neuroinflammation, the therapeutic role of Ago2, and associated miRNA, should be explored in acute or degenerative neuropathological contexts.

## Supplementary Information

**Additional file 1.** Representative confocal images of Ago2 (red) and NRP1 expression (green) by brain endothelial cells under physiological conditions (scale bar 10 μm).

**Additional file 2. Table S1.** List of primary and secondary antibodies, and dilutions used for western blot analysis.

**Additional file 3.** Panel 1. Representative protein bands obtained from western blotting experiments performed in Figs. 1a, e and h (panel a), 2a, c and f (panel b), 3a and f (panel c), 4c (panel d), 5 (panel e) and 6 (panel f). Housekeeping (HK) used were tubulin (50 KDa), actin (42 KDa) and GAPDH (37 KDa).

**Additional file 4.** Panel 1. Representative confocal images of Ago2 (red) expression by astrocytes (GFAP+) and microglia (CD11b+) (green) obtained in the neocortex and hippocampus of mice injected with saline (CTR) or 2 mg/kg lipopolysaccharide (LPS) (scale bar 10 μm). Inserts highlight staining (scale bar 20 μm).

## Data Availability

The datasets used and/or analyzed during the current study are available from the corresponding author on reasonable request.

## Abbreviations

Ago2: Argonaute-2
BafA1: Bafilomycin A1
CD11b: Cluster of differentiation molecule 11b
CREB: CAMP response element-binding protein
EC-CM: Endothelial cell-conditioned media
eNOS: Endothelial nitric oxide synthase
GDNF: Glial-derived neurotrophic factor
GFAP: Glial fibrillary acidic protein
Iba-1: Ionized calcium-binding adaptor molecule-1
IL-1β: Interleukin-1 beta
IL-6: Interleukin-6
iNOS: Inducible nitric oxide synthase
LPS: Lipopolysaccharide
MAP2: Microtubule-associated protein
mRNA: Messenger ribonucleic acid
NG2: Oligodendrocyte precursor cells
NO: Nitric oxide
NOX2: NADPH oxidase 2
NRP1: Neuropilin-1
p38: P38 mitogen-activated protein kinases signaling pathway
p47phox: Neutrophil cytosol factor 1
Pp38: Phosphorylated p38 mitogen-activated protein kinases signaling pathway
PSD-95: Postsynaptic density protein-95
RISC: RNA-induced silencing complex
S100B: S100 calcium-binding protein B
TNF-α: Tumor necrosis factor-alpha
TLR4: Toll-like receptor 4
TRAF6: TNF receptor-associated factor 6
VE-cadherin: Vascular endothelial-cadherin

## References

[1] Daneman R, Prat A (2015). The blood-brain barrier. Cold Spring Harb Perspect Biol.

[2] Varatharaj A, Galea I (2017). The blood-brain barrier in systemic inflammation. Brain Behav Immun.

[3] Machado-Pereira M, Santos T, Bernardino L, Ferreira R (2016). Vascular inter-regulation of inflammation: molecular and cellular targets for CNS therapy. J Neurochem.

[4] Dauphinee SM, Karsan A (2006). Lipopolysaccharide signaling in endothelial cells. Lab Investig.

[5] Akira S, Takeda K (2004). Toll-like receptor signalling. Nat Rev Immunol.

[6] O’Neill L, Sheedy F, McCoy C (2011). MicroRNAs: the fine-tuners of Toll-like receptor signalling. Nat Rev Immunol.

[7] Fabian MR, Sonenberg N, Filipowicz W (2010). Regulation of mRNA translation and stability by microRNAs. Annu Rev Biochem.

[8] Bartel DP (2018). Metazoan microRNAs. Cell.

[9] Liu J, Carmell MA, Rivas FV, Marsden CG, Thomson JM, Song JJ (2004). Argonaute2 is the catalytic engine of mammalian RNAi. Science (80−).

[10] Meister G (2013). Argonaute proteins: functional insights and emerging roles. Nat Rev Genet.

[11] Boon RA, Vickers KC (2013). Intercellular transport of microRNAs. Arterioscler Thromb Vasc Biol.

[12] Arroyo JD, Chevillet JR, Kroh EM, Ruf IK, Pritchard CC, Gibson DF (2011). Argonaute2 complexes carry a population of circulating microRNAs independent of vesicles in human plasma. Proc Natl Acad Sci USA.

[13] Schmitter D, Filkowski J, Sewer A, Pillai RS, Oakeley EJ, Zavolan M (2006). Effects of dicer and argonaute down-regulation on mRNA levels in human HEK293 cells. Nucleic Acids Res.

[14] Martinez NJ, Gregory RI (2013). Argonaute2 expression is post-transcriptionally coupled to microRNA abundance. RNA.

[15] Winter J, Diederichs S (2011). Argonaute proteins regulate microRNA stability: increased microRNA abundance by Argonaute proteins is due to microRNA stabilization. RNA Biol.

[16] Asai T, Suzuki Y, Matsushita S, Yonezawa S, Kiwada H, Nango M (2008). Disappearance of the angiogenic potential of endothelial cells caused by Argonaute2 knockdown. Biochem Biophys Res Commun.

[17] Ye ZL, Huang Y, Li LF, Zhu HL, Gao HX, Liu H (2015). Argonaute 2 promotes angiogenesis via the PTEN/VEGF signaling pathway in human hepatocellular carcinoma. Acta Pharmacol Sin.

[18] Ferreira R, Santos T, Amar A, Gong A, Chen TC, Tahara SM (2014). Argonaute-2 promotes miR-18a entry in human brain endothelial cells. J Am Heart Assoc.

[19] Prud GJ, Glinka Y, Lichner Z, Yousef GM (2016). Neuropilin-1 is a receptor for extracellular miRNA and AGO2/miRNA complexes and mediates the internalization of miRNAs that modulate cell function. Oncotarget.

[20] Dueck A, Ziegler C, Eichner A, Berezikov E, Meister G (2012). MicroRNAs associated with the different human Argonaute proteins. Nucleic Acids Res.

[21] Wu Z, Hofman FM, Zlokovic BV (2003). A simple method for isolation and characterization of mouse brain microvascular endothelial cells. J Neurosci Methods.

[22] Cristóvão AC, Saavedra A, Fonseca CP, Campos F, Duarte EP, Baltazar G (2010). Microglia of rat ventral midbrain recovers its resting state over time in vitro: let microglia rest before work. J Neurosci Res.

[23] Cazareth J, Guyon A, Heurteaux C, Chabry J, Petit-Paitel A (2014). Molecular and cellular neuroinflammatory status of mouse brain after systemic lipopolysaccharide challenge: importance of CCR2/CCL2 signaling. J Neuroinflamm.

[24] Saraiva C, Barata-Antunes S, Santos T, Ferreiro E, Cristóvão AC, Serra-Almeida C (2019). Histamine modulates hippocampal inflammation and neurogenesis in adult mice. Sci Rep.

[25] Danilov CA, Gu Y, Punj V, Wu Z, Steward O, Sch AH (2020). Intravenous delivery of microRNA-133b along with Argonaute-2 enhances spinal cord recovery following cervical contusion in mice. Spine J.

[26] Ferreira R, Xapelli S, Santos T, Silva AP, Cristóvão A, Cortes L (2010). Neuropeptide y modulation of interleukin-1β (IL-1β)-induced nitric oxide production in microglia. J Biol Chem.

[27] Ferreira R, Santos T, Cortes L, Cochaud S, Agasse F, Silva AP (2012). Neuropeptide y inhibits interleukin-1 beta-induced microglia motility. J Neurochem.

[28] Ferreira R, Santos T, Viegas M, Cortes L, Bernardino L, Vieira OV (2011). Neuropeptide Y inhibits interleukin-1β-induced phagocytosis by microglial cells. J Neuroinflamm.

[29] Machado-Pereira M, Santos T, Ferreira L, Bernardino L, Ferreira R (2017). Anti-inflammatory strategy for M2 microglial polarization using retinoic acid-loaded nanoparticles. Mediat Inflamm.

[30] Machado-Pereira M, Santos T, Ferreira L, Bernardino L, Ferreira R (2018). Intravenous administration of retinoic acid-loaded polymeric nanoparticles prevents ischemic injury in the immature brain. Neurosci Lett.

[31] Vestweber D (2008). VE-cadherin: the major endothelial adhesion molecule controlling cellular junctions and blood vessel formation. Arterioscler Thromb Vasc Biol.

[32] Sainson RCA, Johnston DA, Chu HC, Holderfield MT, Nakatsu MN, Crampton SP (2008). TNF primes endothelial cells for angiogenic sprouting by inducing a tip cell phenotype. Blood.

[33] Gopinathan G, Milagre C, Pearce OMT, Reynolds LE, Hodivala-Dilke K, Leinster DA (2015). Interleukin-6 stimulates defective angiogenesis. Cancer Res.

[34] Macvicar BA, Newman EA (2015). Astrocyte regulation of blood flow in the brain. Cold Spring Harb Perspect Biol.

[35] Yang F-M, Zuo Y, Zhou W, Xia C, Hahm B, Sullivan M (2018). sNASP inhibits TLR signaling to regulate immune response in sepsis. J Clin Invest.

[36] Xing C, Li W, Deng W, Ning MM, Lo EH (2018). A potential gliovascular mechanism for microglial activation: differential phenotypic switching of microglia by endothelium versus astrocytes. J Neuroinflamm.

[37] Sánchez C, Díaz-Nido J, Avila J (2000). Phosphorylation of microtubule-associated protein 2 (MAP2) and its relevance for the regulation of the neuronal cytoskeleton function. Prog Neurobiol.

[38] Alberini CM (2009). Transcription factors in long-term memory and synaptic plasticity. Physiol Rev.

[39] Kim E, Sheng M (2004). PDZ domain proteins of synapses. Nat Rev Neurosci.

[40] Wild JRL, Staton CA, Chapple K, Corfe BM (2012). Neuropilins: expression and roles in the epithelium. Int J Exp Pathol.

[41] Bae D, Lu S, Taglienti CA, Mercurio AM (2008). Metabolic stress induces the lysosomal degradation of neuropilin-1 but not neuropilin-2. J Biol Chem.

[42] Reglero-Real N, Pérez-Gutiérrez L, Yoshimura A, Rolas L, Garrido-Mesa J, Barkaway A (2021). Autophagy modulates endothelial junctions to restrain neutrophil diapedesis during inflammation. Immunity.

[43] Dai X, Okon I, Liu Z, Wu Y, Zhu H, Song P (2017). A novel role for myeloid cell-specific neuropilin 1 in mitigating sepsis. FASEB J.

[44] Bielenberg DR, Klagsbrun M (2007). Targeting endothelial and tumor cells with semaphorins. Cancer Metastasis Rev.

[45] Geekiyanage H, Rayatpisheh S, Wohlschlegel JA, Brown R, Ambros V (2020). Extracellular microRNAs in human circulation are associated with miRISC complexes that are accessible to anti-AGO2 antibody and can bind target mimic oligonucleotides. Proc Natl Acad Sci USA.

[46] Schaaf MB, Houbaert D, Meçe O, Agostinis P (2019). Autophagy in endothelial cells and tumor angiogenesis. Cell Death Differ.

[47] Wang D, Zhang Z, O’Loughlin E, Lee T, Houel S, O’Carroll D (2012). Quantitative functions of argonaute proteins in mammalian development. Genes Dev.

[48] González D, Herrera B, Beltrán A, Otero K, Quintero G, Rojas A (2003). Nitric oxide disrupts VE-cadherin complex in murine microvascular endothelial cells. Biochem Biophys Res Commun.

[49] Chan YH, Harith HH, Israf DA, Tham CL (2020). Differential regulation of LPS-mediated VE-cadherin disruption in human endothelial cells and the underlying signaling pathways: a mini review. Front Cell Dev Biol.

[50] Slavin SA, Leonard A, Grose V, Fazal F, Rahman A (2018). Autophagy inhibitor 3-methyladenine protects against endothelial cell barrier dysfunction in acute lung injury. Am J Physiol - Lung Cell Mol Physiol.

[51] Abbott NJ (2002). Astrocyte-endothelial interactions and blood–brain barrier permeability. J Anat.

[52] Chen S-H, Oyarzabal E, Sung Y-F, Chu C-H, Wang Q, Chen S-L (2015). Microglial regulation of immunological and neuroprotective functions of astroglia. Glia.

[53] Brambilla L, Guidotti G, Martorana F, Iyer AM, Aronica E, Valori CF (2016). Disruption of the astrocytic TNFR1-GDNF axis accelerates motor neuron degeneration and disease progression in amyotrophic lateral sclerosis. Hum Mol Genet.

[54] Mazumder A, Bose M, Chakraborty A, Chakrabarti S, Bhattacharyya SN (2013). A transient reversal of miRNA-mediated repression controls macrophage activation. EMBO Rep.

[55] Zhong G, Geller DA, Litwack G (2014). Chapter two—microRNA and human inducible nitric oxide synthase. Nitric oxide.

[56] Leonov G, Shah K, Yee D, Timmis J, Sharp TV, Lagos D (2015). Suppression of AGO2 by miR-132 as a determinant of miRNA-mediated silencing in human primary endothelial cells. Int J Biochem Cell Biol.

[57] Liew FY, Xu D, Brint EK, O’Neill LAJ (2005). Negative regulation of Toll-like receptor-mediated immune responses. Nat Rev Immunol.

[58] Walsh MC, Lee J, Choi Y (2015). Tumor necrosis factor receptor- associated factor 6 (TRAF6) regulation of development, function, and homeostasis of the immune system. Immunol Rev.

[59] Iannucci J, Rao HV, Grammas P (2020). High glucose and hypoxia-mediated damage to human brain microvessel endothelial cells induces an altered, pro-inflammatory phenotype in BV-2 microglia in vitro. Cell Mol Neurobiol.

[60] Maksoud MJE, Tellios V, Xiang YY, Lu WY (2020). Nitric oxide signaling inhibits microglia proliferation by activation of protein kinase-G. Nitric Oxide Biol Chem.

[61] Mannerström B, Paananen RO, Abu-Shahba AG, Moilanen J, Seppänen-Kaijansinkko R, Kaur S (2019). Extracellular small non-coding RNA contaminants in fetal bovine serum and serum-free media. Sci Rep.

[62] Weaver AM, Patton JG (2020). Argonautes in extracellular vesicles: artifact or selected cargo?. Cancer Res.

[63] Hoogland ICM, Houbolt C, van Westerloo DJ, van Gool WA, van de Beek D (2015). Systemic inflammation and microglial activation: systematic review of animal experiments. J Neuroinflamm.

[64] Banks WA, Robinson SM (2010). Minimal penetration of lipopolysaccharide across the murine blood–brain barrier. Brain Behav Immun.

[65] Bode JG, Ehlting C, Haussinger D (2012). The macrophage response towards LPS and its control through the p38MAPK–STAT3 axis. Cell Signal.

[66] Trevelin SC, Shah AM, Lombardin G (2020). Beyond bacterial killing: NADPH oxidase 2 is an immunomodulator. Immunol Lett.

[67] Carbonell T, Gomes AV (2020). MicroRNAs in the regulation of cellular redox status and its implications in myocardial ischemia-reperfusion injury. Redox Biol.

[68] Ranjan R, Lee YG, Karpurapu M, Syed MA, Chung S, Deng J (2015). p47phox and reactive oxygen species production modulate expression of microRNA-451 in macrophages. Free Radic Res.

[69] Wang T, Liu YP, Wang T, Xu BQ, Xu B (2017). ROS feedback regulates the microRNA-19-targeted inhibition of the p47phox-mediated LPS-induced inflammatory response. Biochem Biophys Res Commun.

[70] Bam M, Yang X, Zumbrun EE, Ginsberg JP, Leyden Q, Zhang J (2017). Decreased AGO2 and DCR1 in PBMCs from War Veterans with PTSD leads to diminished miRNA resulting in elevated inflammation. Transl Psychiatry.

[71] Kerckhove M, Tanaka K, Umehara T, Okamoto M, Kanematsu S, Hayashi H (2018). Targeting miR-223 in neutrophils enhances the clearance of *Staphylococcus**aureus* in infected wounds. EMBO Mol Med.

[72] Bellissimo T, Tito C, Ganci F, Sacconi A, Masciarelli S, Di Martino G (2019). Argonaute 2 drives miR-145-5p-dependent gene expression program in breast cancer cells. Cell Death Dis.

[73] Liu X, Meng X, Peng X, Yao Q, Zhu F, Ding Z (2021). Impaired AGO2/miR-185-3p/NRP1 axis promotes colorectal cancer metastasis. Cell Death Dis.

[74] Steiner J, Bernstein HG, Bielau H, Berndt A, Brisch R, Mawrin C (2007). Evidence for a wide extra-astrocytic distribution of S100B in human brain. BMC Neurosci.

[75] Donato R (2001). S100: a multigenic family of calcium-modulated proteins of the EF-hand type with intracellular and extracellular functional roles. Int J Biochem Cell Biol.

[76] Savchenko VL, Nikonenko IR, Skibo GG, McKanna JA (1997). Distribution of microglia and astrocytes in different regions of the normal adult rat brain. Neurophysiology.

[77] Parisi C, Giorgi C, Batassa EM, Braccini L, Maresca G, D’Agnano I (2011). Ago1 and Ago2 differentially affect cell proliferation, motility and apoptosis when overexpressed in SH-SY5Y neuroblastoma cells. FEBS Lett.

[78] Zhang X, Graves P, Zeng Y (2012). Overexpression of human Argonaute2 inhibits cell and tumor growth. Biochim Biophys Acta - Gen Subj.

[79] Adams BD, Claffey KP, White BA (2009). Argonaute-2 expression is regulated by epidermal growth factor receptor and mitogen-activated protein kinase signaling and correlates with a transformed phenotype in breast cancer cells. Endocrinology.

[80] Cheng N, Li Y, Han ZG (2013). Argonaute2 promotes tumor metastasis by way of up-regulating focal adhesion kinase expression in hepatocellular carcinoma. Hepatology.

[81] Liddelow SA, Guttenplan KA, Clarke LE, Bennett FC, Bohlen CJ, Schirmer L (2017). Neurotoxic reactive astrocytes are induced by activated microglia. Nature.

[82] Arai K, Matsuki N, Ikegaya Y, Nishiyama N (2001). Deterioration of spatial learning performances in lipopolysaccharide-treated mice. Jpn J Pharmacol.

[83] Noh H, Jeon J, Seo H (2014). Systemic injection of LPS induces region-specific neuroinflammation and mitochondrial dysfunction in normal mouse brain. Neurochem Int.

[84] Maria E, Costanzi M, Saraulli D, Scardigli R, Barbato C, Cogoni C (2010). RISC activity in hippocampus is essential for contextual memory. Neurosci Lett.

