# Supplementary material for: Argonaute-2 protects the neurovascular unit from damage caused by systemic inflammation
Source: J Neuroinflammation. 2022 Jan 6;19:11. doi: 10.1186/s12974-021-02324-7 (PMC8740421; doi:10.1186/s12974-021-02324-7)

Additional File 1: Representative confocal images of Ago2 (red) and NRP1 expression (green) by brain endothelial cells under physiological conditions (scale bar 10 μm).

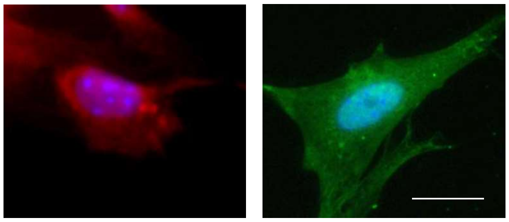

Supplement: Supplementary file 1 — Additional file 1. Representative confocal images of Ago2 (red) and NRP1 expression (green) by brain endothelial cells under physiological conditions (scale bar 10 μm). [file 12974_2021_2324_MOESM1_ESM.pdf]
