# Supplementary material for: Argonaute-2 protects the neurovascular unit from damage caused by systemic inflammation
Source: J Neuroinflammation. 2022 Jan 6;19:11. doi: 10.1186/s12974-021-02324-7 (PMC8740421; doi:10.1186/s12974-021-02324-7)
Supplement: Supplementary file 2 — Additional file 2. Table S1. List of primary and secondary antibodies, and dilutions used for western blot analysis. [file 12974_2021_2324_MOESM2_ESM.pdf]

Additional File 2: List of primary and secondary antibodies, and dilutions used for western blot analysis.

Primary antibody	Abbreviation	Company	Host	Dilution
Argonaute-2	Ago2	Cell Signaling, MA, USA	Rabbit	1:500
Neuropilin-1	NRP1	Origene, MD, USA	Rabbit	1:500
Vascular endothelial-cadherin	VE-cadherin	St John’s Laboratory, London, UK	Rabbit	1:1000
Glial fibrillary acidic protein	GFAP	BD Biosciences, CA, USA	Mouse	1:1000
Glial cell line-derived neurotrophic factor	GDNF	Santa Cruz Biotechnology, CA, USA	Rabbit	1:250
TNF receptor-associated factor 6	TRAF6	Thermo Fisher Scientific, MA, USA	Rabbit	1:1000
Cluster of differentiation molecule 11b	CD11b	St John’s Laboratory, London, UK	Rabbit	1:500
Phosphorylated p38 mitogen-activated protein kinase	Pp38	Cell Signaling, MA, USA	Rabbit	1:1000
p38 mitogen-activated protein kinase (total levels)	Total p38	Cell Signaling, MA, USA	Rabbit	1:1000
Endothelial nitric oxide synthase	eNOS	BD Biosciences, CA, USA	Mouse	1:1000
Inducible nitric oxide synthase	iNOS	BD Biosciences, CA, USA	Rabbit	1:1000
NADPH oxidase 2	NOX2	BD Biosciences, CA, USA	Mouse	1:1000
NADPH oxidase cytosolic protein p47phox	p47phox	St John’s Laboratory, London, UK	Rabbit	1:1000
Ionized calcium-binding adapter molecule-1	Iba-1	Santa Cruz Biotechnology, CA, USA	Mouse	1:200
S100 calcium-binding protein B	S100B	BD Biosciences, CA, USA	Mouse	1:5000
cAMP-response element binding protein	CREB	Cell Signaling, MA, USA	Rabbit	1:1000
Microtubule-associated protein 2	MAP2	Santa Cruz Biotechnology, CA, USA	Rabbit	1:1000
Postsynaptic density protein 95	PSD-95	Merck Millipore, Darmstadt, Germany	Mouse	1:1000

Secondary antibody	Abbreviation	Company	Host	Dilution
Anti-rabbit	Anti-rabbit	Santa Cruz Biotechnology, CA, USA	Goat	1:5000
Anti-mouse	Anti-mouse	Santa Cruz Biotechnology, CA, USA	Goat	1:10000

Housekeeping targets	Abbreviation	Company	Host	Dilution
Tubulin	Tubulin	Sigma, MO, USA	Mouse	1:5000
Actin	Actin	BD Biosciences, CA, USA	Mouse	1:5000
Glyceraldehyde 3-phosphate dehydrogenase	GAPDH	Merck Millipore, Darmstadt, Germany	Mouse	1:5000
