# Supplementary material for: Argonaute-2 protects the neurovascular unit from damage caused by systemic inflammation
Source: J Neuroinflammation. 2022 Jan 6;19:11. doi: 10.1186/s12974-021-02324-7 (PMC8740421; doi:10.1186/s12974-021-02324-7)

Additional File 3: Representative protein bands obtained from western blotting experiments. Housekeeping (HK) used were tubulin (50 KDa), actin (42 KDa) and GAPDH (37 KDa).

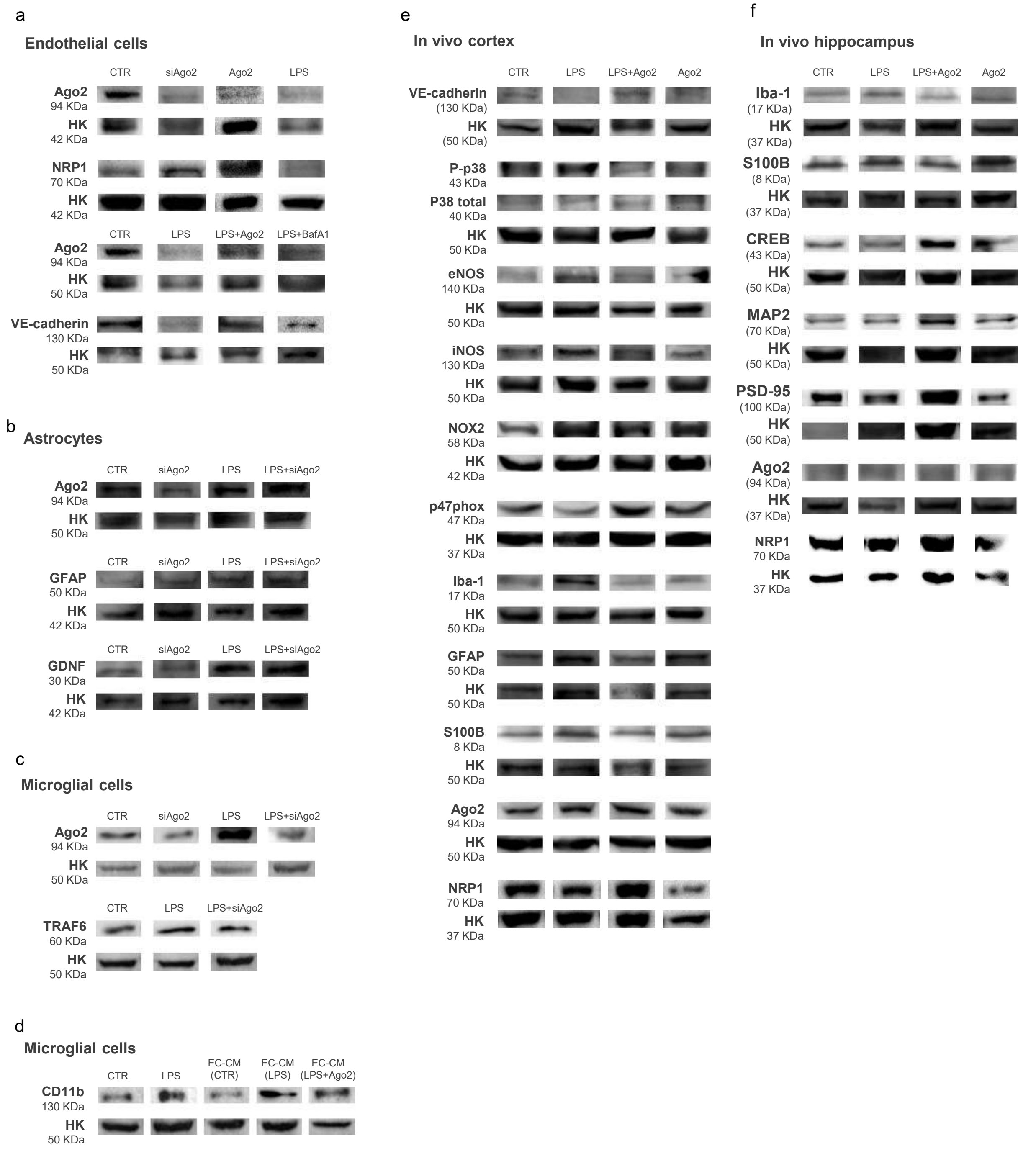

Supplement: Supplementary file 3 — Additional file 3. Panel 1. Representative protein bands obtained from western blotting experiments performed in Figs. 1a, e and h (panel a), 2a, c and f (panel b), 3a and f (panel c), 4c (panel d), 5 (panel e) and 6 (panel f). Housekeeping (HK) used were tubulin (50 KDa), actin (42 KDa) and GAPDH (37 KDa). [file 12974_2021_2324_MOESM3_ESM.pdf]
