# Supplementary material for: Argonaute-2 protects the neurovascular unit from damage caused by systemic inflammation
Source: J Neuroinflammation. 2022 Jan 6;19:11. doi: 10.1186/s12974-021-02324-7 (PMC8740421; doi:10.1186/s12974-021-02324-7)

Additional File 4: Representative confocal images of Ago2 (red) expression by astrocytes (GFAP+) and microglia (CD11b+) (green) obtained in the neocortex and hippocampus of mice injected with saline (CTR) or 2 mg/kg lipopolysaccharide (LPS) (scale bar 10 μm). Inserts highlight staining (scale bar 20 μm).

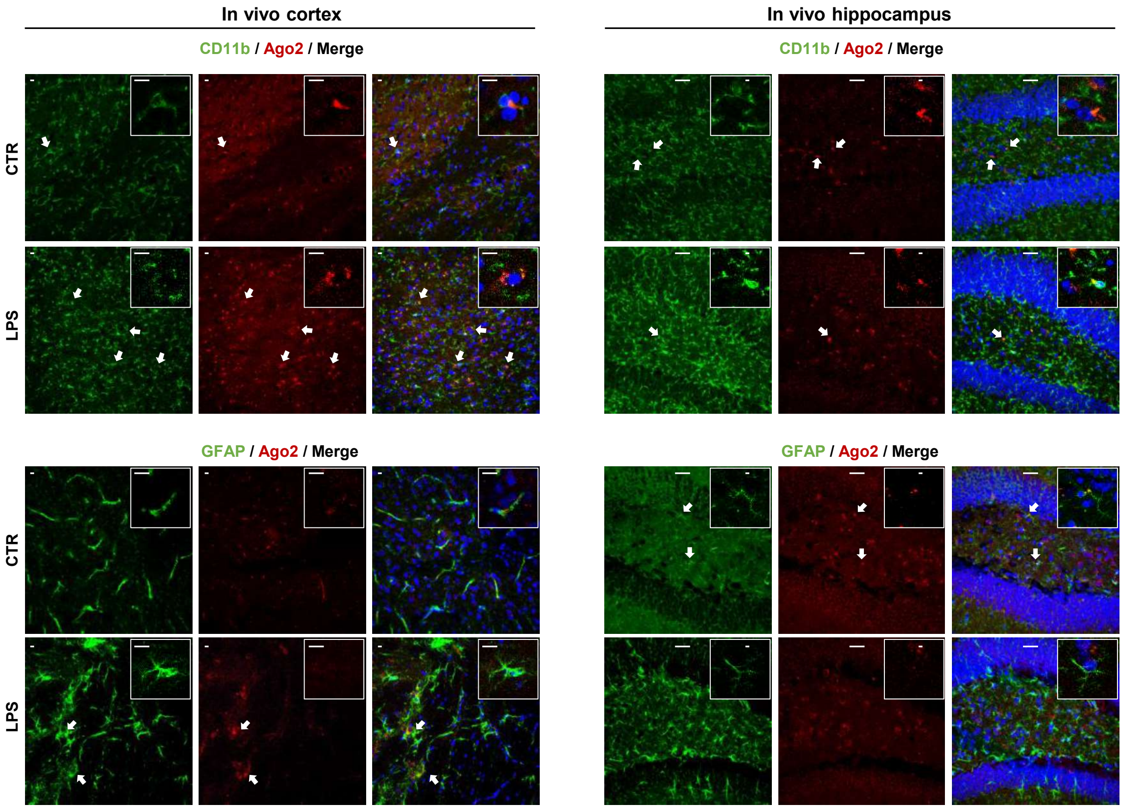

Supplement: Supplementary file 4 — Additional file 4. Panel 1. Representative confocal images of Ago2 (red) expression by astrocytes (GFAP+) and microglia (CD11b+) (green) obtained in the neocortex and hippocampus of mice injected with saline (CTR) or 2 mg/kg lipopolysaccharide (LPS) (scale bar 10 μm). Inserts highlight staining (scale bar 20 μm). [file 12974_2021_2324_MOESM4_ESM.pdf]
